# HER2 reduces breast cancer radiosensitivity by activating focal adhesion kinase *in vitro* and *in vivo*

**DOI:** 10.18632/oncotarget.9870

**Published:** 2016-06-07

**Authors:** Jing Hou, Zhirui Zhou, Xingxing Chen, Ruping Zhao, Zhaozhi Yang, Na Wei, Qing Ni, Yan Feng, Xiaoli Yu, Jinli Ma, Xiaomao Guo

**Affiliations:** ^1^ Department of Breast surgery, Guizhou Provincial People's Hospital, Guiyang 550002, Guizhou Province, China; ^2^ Department of Radiation Oncology, Fudan University Shanghai Cancer Center, Shanghai 200032, China; ^3^ Department of Oncology, Shanghai Medical College, Fudan University, Shanghai 200032, China; ^4^ Department of Radiation Oncology, Hangzhou Cancer Hospital, Hangzhou 310006, Zhejiang, China

**Keywords:** breast cancer, radiosensitivity, HER2, focal adhesion kinase, anoikis

## Abstract

Growing evidence has demonstrated that human epidermal growth factor receptor 2 (HER2) is involved in the radiation response to breast cancer. However, the underlying mechanism remains elusive. Therefore, we investigated if HER2 overexpression is associated with radiosensitivity of breast cancer. We constructed breast cancer cell lines differing in HER2 expression by transducing HER2 cDNA or short hairpin RNA against HER2. We then assessed the radiosensitivity and investigated the potential mechanism by using cell proliferation assay, cell adhesion assays, anoikis assays, colony formation assays, and western blotting analyses. We found that HER2 introduction in breast cancer cell lines MCF-7 (low HER2 expression) and MDA-MB-231 (HER2 is not expressed) promoted cell proliferation and invasion and enhanced cell adhesion and resistance to anoikis. Moreover, HER2 reduced radiosensitivity in these two cells compared with the corresponding control. The opposite results were observed when HER2 was silenced in breast cancer cell lines ZR-7530 and SK-BR-3 (both cells with high expression of HER2) using HER2 shRNA. In addition, animal experiment results showed HER2 could enhance the radioresistance of xenograft tumors. Further studies showed HER2 promoted the phosphorylation of focal adhesion kinase (Fak) and thereby up-regulated the expression of proteins associated with the epithelial-to-mesenchymal transition such as Claudin-1, ZO-1, and ZEB-1. The inhibition of Fak activity using the Fak inhibitor (PF-562281) restored the radiosensitivity in HER2-overexpressing cells. In conclusion, HER2 reduces the radiosensitivity of breast cancer by activating Fak *in vitro* and *in vivo*. Fak might be a potential target for the radiosensitization of HER2-overexpressed breast cancer.

## INTRODUCTION

Over the past 30 years, the mortality of breast cancer in females has shown a gradual uptrend in China, which makes breast cancer a crucial cause of death in females [1]. Radiotherapy plays an important role in the comprehensive treatment of breast cancer [2, 3]. Postoperative radiotherapy could reduce the local and regional recurrence rate of lymph nodes by approximately two-thirds. Breast conserving surgery plus radiotherapy could decrease the local recurrence rate from 18%–35% to 2%–10% [4, 5]. However, some patients still relapse, suggesting that these tumors might be insensitive to radiotherapy. Breast cancer is a highly heterogeneous disease that could be divided into four molecular subtypes according to gene expression profiles: luminal subtype, HER2-overexpressing subtype, basal-like subtype, and normal breast-like subtype [6, 7]. Accumulating literature has demonstrated that the local recurrence varies in different subtypes of breast cancer after comprehensive treatments, indicating that the molecular subtype acts as a prognostic factor for breast cancer [8–10].

Genomic amplification of the HER2 gene and/or overexpression of its product occur in 20%–30% of all types of breast cancers. HER2 overexpression is a hallmark in this category of breast cancer, and HER2 status is the determinant of the treatment option [11]. HER2 overexpression is correlated with aggressive tumor growth and an increased recurrence of breast cancer [8–10]. The approval of trastuzumab in 1998 was a landmark in the era of “targeted therapy” because trastuzumab has significantly improved patient outcomes of HER2-overexpressing breast cancer subtype and paved the way for the advent of “tailored therapy” for breast cancer [9, 12, 13]. However, the efficiency of trastuzumab, to some extent, reduces the concerns regarding HER2-induced radioresistance in breast cancer. Exposure of tumor cells to ionizing radiation results in immediate activation of the EGFR family, and repeated radiation exposures of 2 Gy leads to increased EGFR expression. Similar phenomena were also observed in HER2. These findings suggested a potential biological role of the HER2 status in the response to radiation [14]. A higher local recurrence rate in HER2-positive patients after surgery and radiotherapy also implies that these tumors are radioresistant. However, few studies have focused on the association between HER2 and radiosensitivity of breast cancer. The molecular mechanisms of the governing tumor resistance of HER2 during radiotherapy remains elusive. To further improve HER2-targeted therapy, it is essential to investigate if and how HER2 influences the radiosensitivity of breast cancer.

Non-receptor focal adhesion kinase (Fak) is a major protein of the focal adhesion complex that integrates signals from growth factors and integrin to control cell adhesion, migration and invasion [15, 16]. Fak is up-regulated in various epithelial cancers, including breast cancer. Several studies indicate that Fak is overexpressed in HER2-positive breast cancer compared with HER2-negative breast cancer [17]. However, the relationship between Fak and HER2 is not well studied. Several investigations found that HER2 and Fak share many common characteristics in regulating cell invasiveness, proliferation, stemness [15, 16]. Therefore, we hypothesize that Fak is activated by HER2 and acts as a downstream target of HER2 to execute cell proliferation and adhesion and might be involved in the modification of breast cancer radiosensitivity.

## RESULTS

### HER2 promotes breast cancer cell proliferation

**Figure 1:**
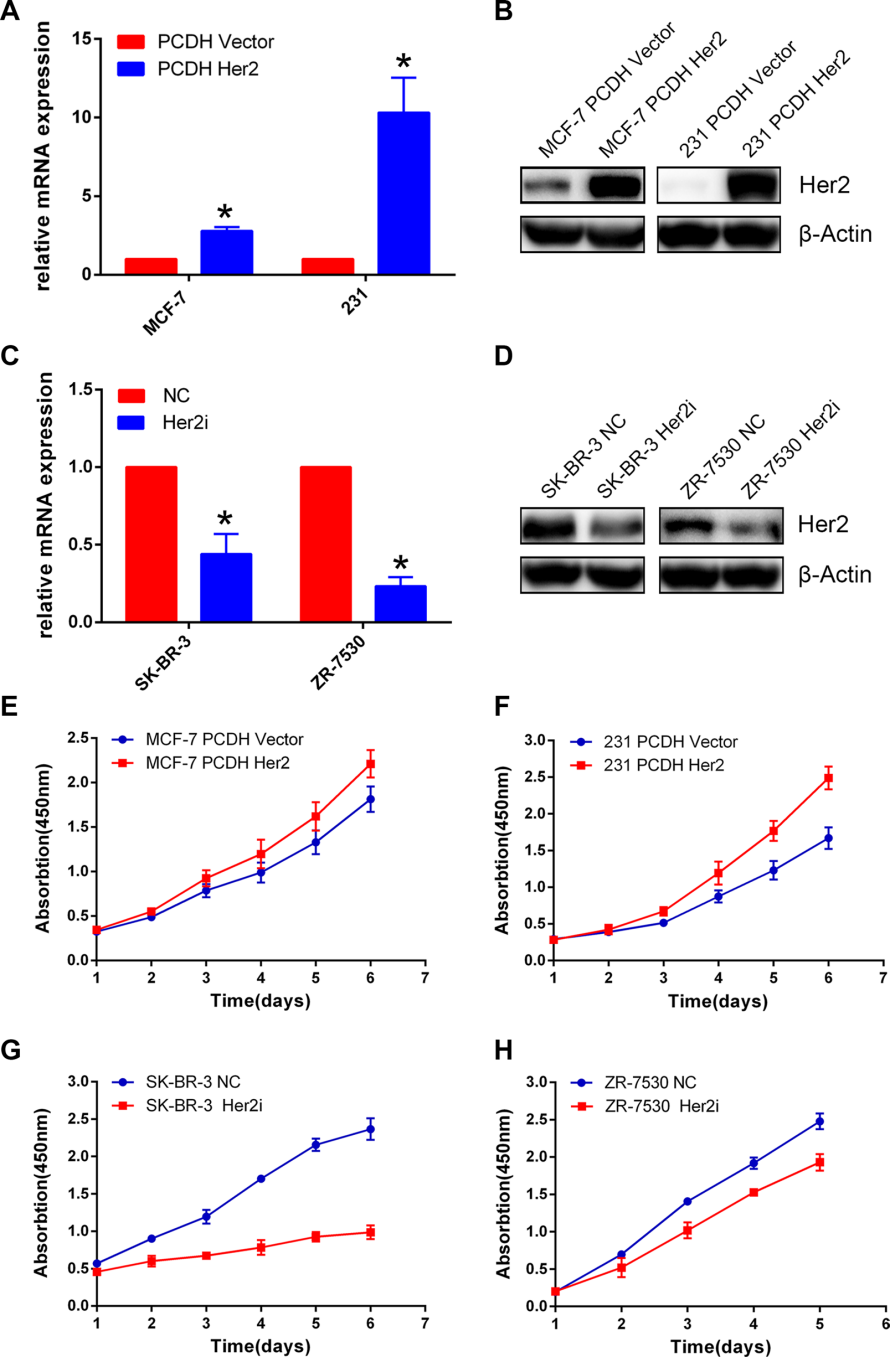
HER2 promotes breast cancer cell proliferation (**A**–**D**) Real-time quantitative PCR (A, C) and Western blot (B, D) validation of cell construction with different HER2 expression levels. β-Actin was used as an internal control. The relative level of HER2 mRNA expression in control cells was set to 1. The relative levels of HER2 mRNA expression of MCF-7 Her2 and MDA-MB-231 Her2 were approximately 3- to 10-fold of the control (A); the relative levels of HER2 mRNA expression of SK-BR-3 Her2i and ZR-7530 Her2i were approximately 0.2- to 0.4-fold of the control (C). (**E**, **F**) HER2 overexpression facilitates the proliferation of breast cancer cells compared with control cells (MCF-7 and MDA-MB-231). (**G**), (**H**) HER2 silencing inhibits the proliferation of breast cancer cells (SK-BR-3 and ZR-7530). The error bars represent 95% confidence intervals (CIs). NC: negative control. *represent *p* value < 0.05.

HER2 is not expressed in MDA-MB-231(231) cells, and MCF-7 has low HER2 expression, whereas ZR-7530, SK-BR-3 cells have high expression of HER2. We constructed MDA-MB-231 for the overexpression of HER2 and ZR-7530 and SK-BR-3 for the silencing of HER2. To establish these cell lines, lentiviruses carrying HER2 cDNA or shRNA against HER2 were generated and used to infect target cells (the corresponding control cells were infected with empty vector or scrambled shRNA viruses). The establishment of cell lines was confirmed by q-PCR and Western blotting (Figure 1A–1D). The relative levels of HER2 mRNA expression of MCF-7 Her2 and MDA-MB-231 Her2 were approximately 3- to 10-fold of the control (A); the relative levels of HER2 mRNA expression of SK-BR-3 Her2i and ZR-7530 Her2i were approximately 0.2- to 0.4-fold of the control (C). We then performed CCK-8 assays to measure the proliferation of each pair of cells. We found that HER2 overexpression promoted proliferation of MCF-7 and MDA-MB-231 cells compared with their corresponding control cells. The opposite results were observed in the ZR-7530 and SK-BR-3 cells when HER2 was silenced (Figure 1E–1H).

### HER2 overexpression reduces radiosensitivity of breast cancer *in vitro* and *in vivo*

**Figure 2:**
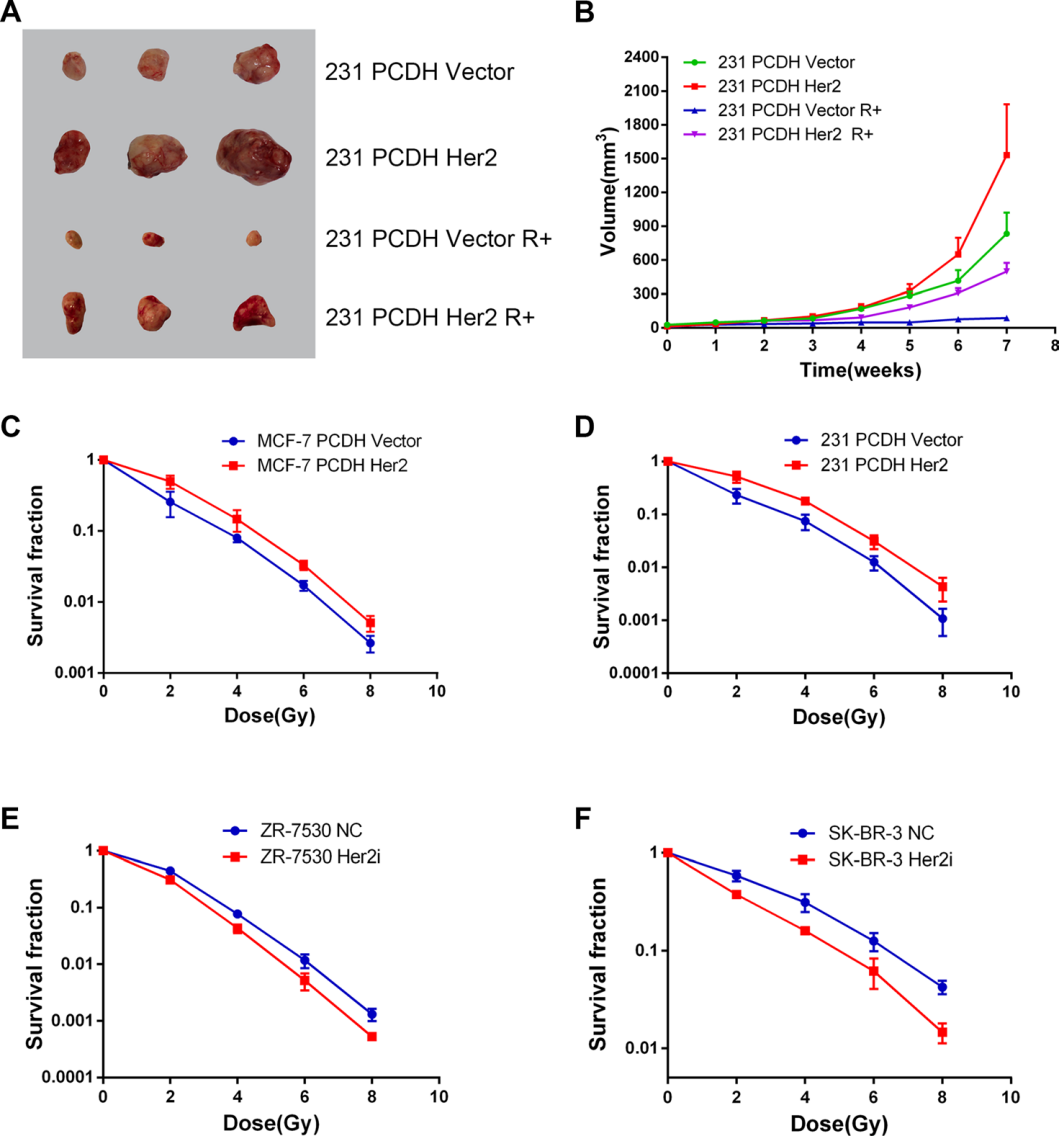
HER2 overexpression reduces radiosensitivity of breast cancer *in vitro* and *in vivo* (**A**, **B**) Decreased radiosensitivity of HER2-overexpressing xenograft tumors in nude mice. Nude mice injected with 231 PCDH HER2 cells developed larger tumor volumes than the mice injected with control cells (mean ± SEM: 1531 ± 452 mm^3^ vs. 834 ± 186 mm^3^; *p* < 0.05). Growth of the tumors was delayed in both groups after irradiation, although the delayed growth was more significant in the mice injected with 231 PCDH vector cells (mean ± SEM: 498 ± 76 mm^3^ vs. 87 ± 24 mm^3^; *p* < 0.05); (**C**, **D**) Survival curves of breast cancer cells after different doses of X-ray irradiation. MCF-7 PCDH HER2 cells and 231 PCDH HER2 cells were less sensitive to radiation compared with their corresponding control cells in the dose range of 2 to 8 Gy; (**E**, **F**) ZR-7530 HER2i and SR-BR-3 HER2i cells were more sensitive to radiation compared with their corresponding control cells. The error bars represent 95% confidence intervals (CIs).

We performed clonogenic assays to test the effect of HER2 on breast cancer cell radiosensitivity. The survival curve showed that MCF-7 PCDH HER2 and 231 PCDH HER2 were less sensitive to radiation compared with their corresponding control cells in the dose range of 2 Gy to 8 Gy (Figure 2C and 2D). ZR-7530 HER2i and SR-BR-3 HER2i cells were more sensitive to radiation compared with their corresponding control cells (Figure 2E and 2F). To determine if HER2 could reduce the sensitization of breast cancer cells to radiation *in vivo*, we injected 231 PCDH HER2 and 231 PCDH vector cells into mice. The results showed that the mice injected with 231 PCDH HER2 cells developed larger tumor volumes compared with the mice injected with control cells (mean ± SEM: 1531 ± 452 mm^3^ vs. 834 ± 186 mm^3^; *p* < 0.05). After irradiation, the growth of the tumors was delayed in both groups. However, the delayed growth was more significant in the mice injected with 231 PCDH vector cells (mean ± SEM: 498 ± 76 mm^3^ vs. 87 ± 24 mm^3^; *p* < 0.05) (Figure 2A and 2B).

### HER2 enhances cell adhesion and anoikis resistance of breast cancer cells

**Figure 3:**
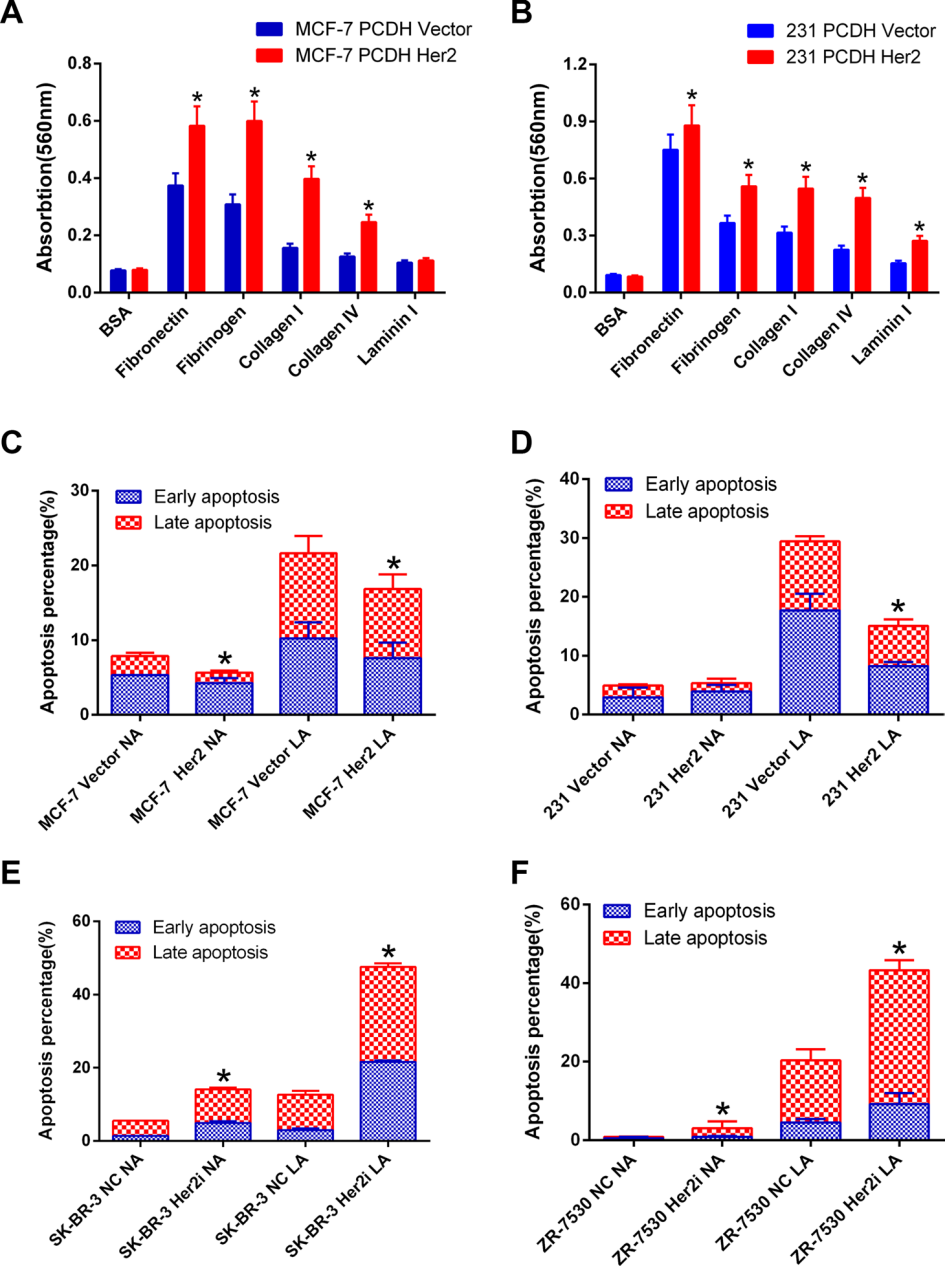
HER2 enhances cell adhesion and anoikis resistance of breast cancer cells (**A**) The adhesion of MCF-7 PCDH HER2 cells was significantly enhanced to fibronectin, fibrinogen, collagen I, and collagen IV compared with the control cells; however, there was no obvious difference for laminin I; (**B**) HER2 overexpression in MDA-MB-231 cells significantly enhanced cell adhesion to fibronectin, followed by fibrinogen, collagen I, collagen IV, and laminin I. (**C**, **D**) The apoptotic percentage of cells overexpressing HER2 is less than their corresponding control cells under low-attachment conditions (mean ± SEM: 17.3% 2.5% vs. 21.8% 2.6%; 15.7% ± 0.5% vs. 29.7% ± 0.6%; *p* < 0.05 in MCF-7 and MDA-MB-231, respectively); (**E**, **F**) The percentage of apoptosis was higher in SK-BR-3 and ZR-7530 cells cultured under low-attachment condition after HER2 was knocked down compared with their parental cells (mean ± SEM: 43.7.3% 0.5% vs. 16.3% 0.6%; 41.6% ± 1.3% vs. 20.2% ± 1.2%; *p* < 0.05 in SK-BR-3 and ZR-7530, respectively). NA: normal-attachment. LA: low-attachment. The error bars represent 95% confidence intervals (CIs). *represent *p* value < 0.05.

We performed cell adhesion assays using ECM-coated plates to detect the adhesion ability of MCF-7 PCDH HER2 and 231 PCDH HER2 cells and their corresponding control cells in five different matrices (bovine serum albumin served as a negative control). The adhesion of MCF-7 PCDH HER2 cells was significantly enhanced to fibronectin, fibrinogen, collagen I, and collagen IV compared with their control cells (*p* < 0.05). However, there was no obvious difference for laminin I (*p* > 0.05) (Figure 3A). HER2 overexpression in MDA-MB-231 cells significantly enhanced cell adhesion to fibronectin, followed by fibrinogen, collagen I, collagen IV, and laminin I (*p* < 0.05) (Figure 3B).

The resistance to anoikis is a hallmark of metastatic cells. Cells lose adhesion to other cells or to the matrix at the beginning of invasion and metastasis, and cells that lose adhesion are subject to various stress, leading to cell apoptosis, namely anoikis. We determined the level of apoptosis in cells after losing cell adhesion. In MCF-7 PCDH HER2 and 231 PCDH HER2 cells, there was no significant difference in the baseline of apoptosis under normal culture conditions compared with their corresponding control cells (*p* > 0.05). The percentage of apoptosis increased in all cells after 24–48 h of suspension culture on ultralow-attachment plates. However, the apoptotic percentage of cells overexpressing HER2 was less than their corresponding control cells (mean ± SEM: 17.3% 2.5% vs. 21.8% 2.6%; 15.7% ± 0.5% vs. 29.7% ± 0.6%, *p* < 0.05 in MCF-7 and MDA-MB-231, respectively), indicating that HER2 overexpressed cells were more resistant to anoikis (Figure 3C and 3D). The opposite results were obtained when HER2 was silenced, in which the percentage of apoptosis was higher both in SK-BR-3 and ZR-7530 cells after HER2 was knocked down (mean ± SEM: 43.7.3% 0.5% vs. 16.3% 0.6% and 41.6% ± 1.3% vs. 20.2% ± 1.2%, *p* < 0.05, in SK-BR-3 and ZR-7530, respectively) when cells were cultured under either low-attachment or normal-attachment conditions (Figure 3E and 3F).

### HER2 promotes Fak phosphorylation

**Figure 4:**
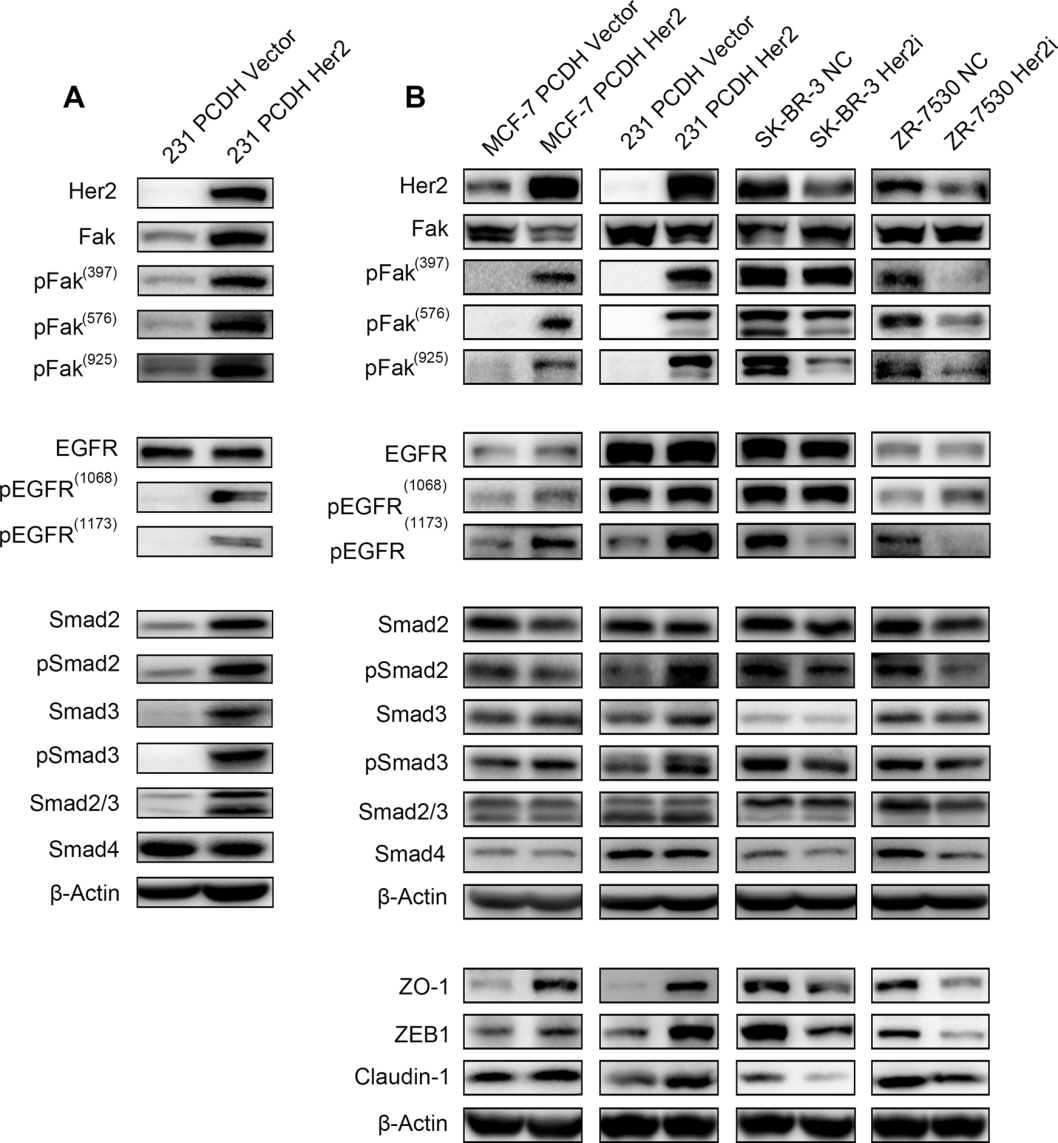
HER2 promotes Fak phosphorylation Immunoblot analysis of Fak and Fak-related molecules (**A**) Fak, pFak (Y397, Y576, and Y925), EGFR, pEGFR, Smad, and pSmad were measured by western blotting with the corresponding antibodies *in vivo* (the protein sample was prepared with tumor tissue from mice injected with 231 PCDH HER2 cells and their corresponding control cells). (**B**) Fak, pFak (Y397, Y576, and Y925), EGFR, pEGFR, Smad, pSmad, ZO-1, ZEB1, and Claudin-1 were measured by Western Blotting with the corresponding antibodies *in vitro*. β-Actin was used as the loading control.

We measured the expression of total Fak protein and the phosphorylation levels of Fak when HER2 was overexpressed or silenced *in vivo* and *in vitro*. First, we extracted the protein lysis from the xenograft tumors of 231 PCDH HER2 cells and their corresponding control cells. Fak phosphorylation (Y397, Y576 and Y925) increased dramatically, and phosphorylated EGFR also increased (Figure 4A). Several studies suggested that Fak plays an important role in TGF-β induced EMT. Therefore, related molecules were detected in our research, and we found that Smad2, Smad3 and their phosphorylated counterparts, which belong to the TGF-β family, were all increased. Smad4 did not show a significant change (Figure 4A). We then detected the expression of these molecules *in vitro*. Compared with the respective controls, MCF-7 PCDH HER2 and 231 PCDH HER2 cells displayed slightly lower Fak protein expression levels but markedly higher phosphorylated Fak (pFak, including Y397, Y576, and Y925) levels; SK-BR-3 HER2i cells showed slightly higher Fak expression with no major change in pFak (Y397) level but marked decreases in pFak (Y576 and Y925) levels; 7530 HER2i cells showed little change in Fak expression and marked decreases in pFak (Y397, Y576, and Y925) levels, indicating that HER2 could promote the phosphorylation of Fak protein (Figure 4B). The Western blot results showed that the EGFR expression levels increased slightly, and the EGFR phosphorylation levels also increased at two major phosphorylation sites (Y1068 and Y1173) in MCF-7 and 231 cells upon HER2 overexpression compared with the respective controls. In contrast, the EGFR expression levels decreased slightly, and the EGFR phosphorylation level also decreased at Y1173 in the SK-BR-3 and 7530 cells upon HER2 silencing. There were no major changes in the EGFR phosphorylation level at Y1068 in the SK-BR-3 cells. The opposite result was obtained at Y1173 from 7530 cells, wherein the EGFR phosphorylation level increased (Figure 4B). We tested the Smads expression. Smad2, p-Smad2, Smad3 and Smad4 did not show significant changes in these 4 cell lines. However, pSmad3 markedly increased in MCF-7 PCDH HER2 and 231 PCDH HER2 cells compared with the controls. In SK-BR-3 HER2i and ZR-7530 HER2i, pSmad3 decreased notably when HER2 was knocked down (Figure 4B). We detected the expression of EMT-related proteins, such as ZEB1, ZO-1 and Claudin-1, and these molecules showed similar changes as HER2. Given these findings, HER2 promotes EGFR phosphorylation and Smad3 phosphorylation, which contribute to Fak phosphorylation leading to EMT (Figure 4B).

### Fak inhibitor induced the radiosensitivity in HER2 overexpressing breast cancer cells

**Figure 5:**
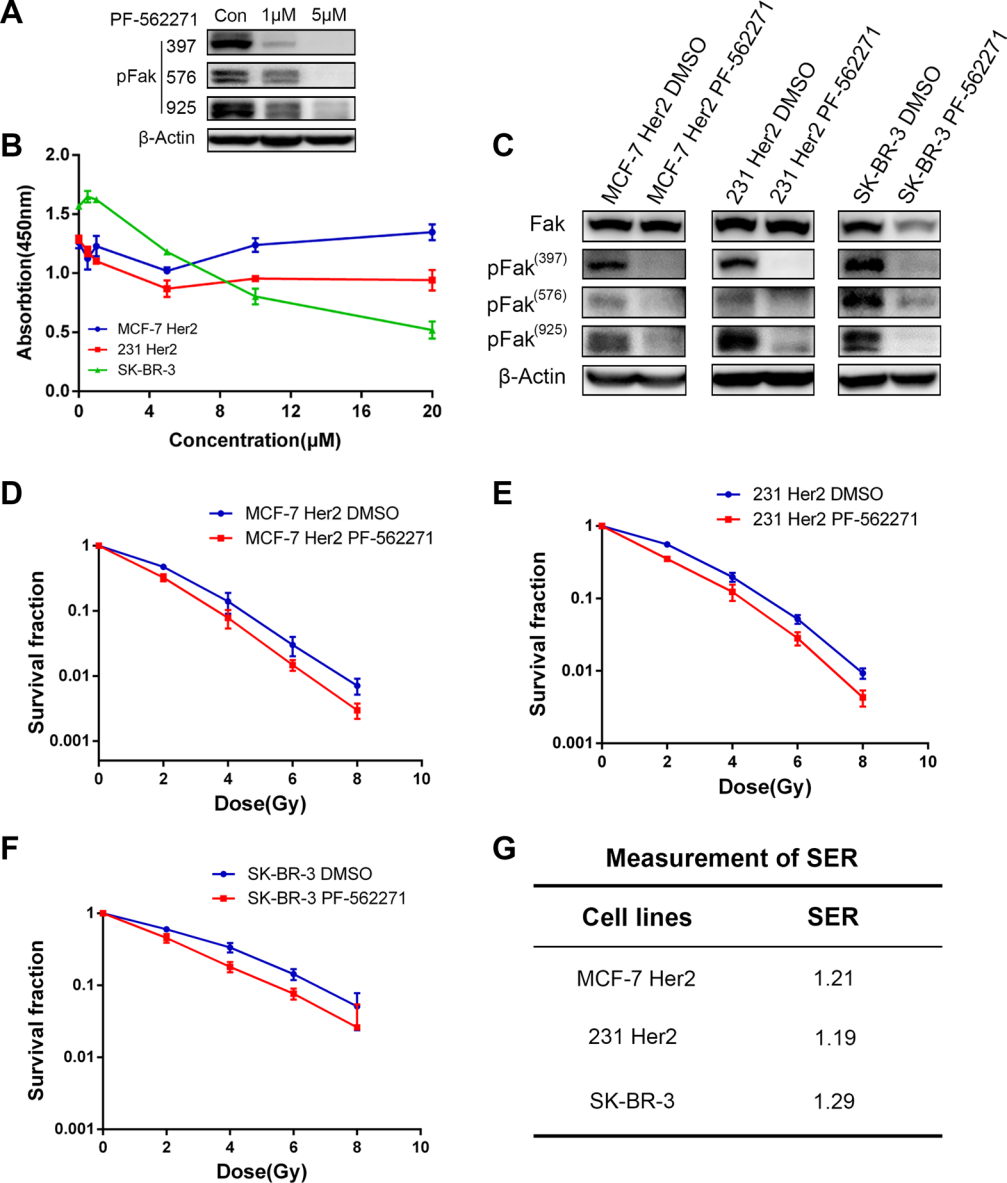
Fak inhibitor induced the radiosensitivity in HER2 overexpressing breast cancer cells (**A**) Immunoblot analysis of pFak after 231 PCDH HER2 cells and their corresponding control cells were treated with PF-562271(1 μM, 5 μM). (**B**) The effects of different concentrations of PF-562271 on the viability of breast cancer cells; (**C**) Changes in Fak and its phosphorylated protein expression in breast cancer cells after 24 h of PF-562271 treatment; (**D**–**G**) PF-562271 induced radiosensitivity in HER2-overexpressing cells. The sensitization enhancement ratio (SER) of PF-562271 in MCF-7 Her 2, MDA-MB-231 Her2 and SK-BR-3 was 1.21, 1.19 and 1.29, respectively. The error bars represent 95% confidence intervals (CIs).

We used a Fak inhibitor (PF-562271) to treat the HER2-overexpressing cell lines and examined the changes in cell radiosensitivity. We first determined the half-maximal inhibitory concentration (IC50) of PF-562271 for MCF-7 PCDH HER2 cells and 231 PCDH HER2 cells and the respective controls after 48 h of treatment. The results showed that PF-562271 had higher IC50 values for MCF-7 PCDH HER2 and 231 PCDH HER2 cells than for the respective controls (data were not shown). PF-562271 was applied at 1 μM and 5 μM in 231 PCDH HER2 cells for 24 hr and pFak was inhibited compared with the DMSO control at 1 μM (Figure 5A). We then treated MCF-7 PCDH HER2, 231 PCDH HER2 and SK-BR-3 cells using different concentrations of PF-562271 and assessed the effect of PF-562271 on cell viability by a CCK-8 assay after 24 h of treatment. We found that 24-h PF-562271 treatment had little effect on the viability of MCF-7 PCDH HER2 and 231 PCDH HER2 cells. However, SK-BR-3 cells exhibited a high sensitivity to the drug concentration. The effect of PF-562271 on the viability of the three cell lines showed little change with a drug concentration lower than 5 μM (Figure 5B). Considering the effect of different concentrations of PF-562271 on cell viability, Fak protein expression and the IC50 values, we selected 1.0 μM PF-562271 treating cells for 24 h in the following experiments. We then examined Fak protein expression in MCF-7 PCDH HER2, 231 PCDH HER2 and SKBR-3 cells after 24 h of treatment with 1 μM PF-562271 (DMSO as control). PF-562271 showed little effect on the total Fak protein but significantly inhibited Fak (Y397, Y576, and Y925) phosphorylation (Figure 5C). We treated cells with the same concentration of PF-562271 for 24 h and then preformed clonogenic assays. The survival advantage of MCF-7 PCDH HER2 and 231 PCDH HER2 cells resulting from HER2 overexpression was reduced to similar levels as the MCF-7 PCDH vector and 231 PCDH vector cells. The response of SK-BR-3 cells to irradiation upon Fak inhibition was similar to the response upon HER2 silencing, indicating that the Fak inhibitor could sensitize breast cancer cells (Figure 5D, 5E and 5F). Furthermore, we calculated the sensitization enhancement ratio (SER) of PF-562271, and the SER values for MCF-7 PCDH HER2, 231 PCDH HER2, and SK-BR-3 cells were 1.21, 1.19, and 1.29, respectively (Figure 5G).

## DISCUSSION

In our study, we determined that HER2 overexpression is linked to radioresistance of breast cancer via the Fak-mediated pathway *in vitro* and *in vivo*. Several clinical studies also show an unfavorable prognosis and poor treatment response in patients with HER2 amplification [8–10, 12, 18]. Although HER2 targeted therapy improves the prognosis of patients with HER2 amplification, HER2 targeted therapy resistance is common in clinical practice [12, 13, 19]. Therefore, it is extremely meaningful to improve radiosensitivity in breast cancer patients with HER2 amplification. In our studies, we observed that a Fak inhibitor (PF-562271) reverses the radioresistant phenotype of the HER2-overexpressed cell lines.

The mechanism of how HER2 increases breast cancer radioresistance remains elusive. Some studies show that HER2 could trigger NF-κB induced radioresistance and that NF-κB could bind to the HER2 promoter, which would lead to the expression of HER2 [20]. Another study suggests that HER2 induced radioresistance might be related to breast cancer stem cells [21]. HER2+/CD44+/CD24–/^low^ cells separated from radioresistant MCF-FIR showed stronger invasive potential [21]. In a similar study, CD44+/CD24–/^low^ also showed greater radioresistance [22]. Circulating tumor cells with EMT property were detected in metastatic breast cancer patients with HER2 amplification [23]. HER2 could be selectively expressed in breast cancer stem cells, which suggests its relationship with cancer stem cells. β-catenin is a vital molecule in the EMT, and β-catenin is involved in HER2-induced invasion and migration in breast cancer [24, 25]. Therefore, HER2 might increase radioresistance in breast cancer via EMT [26]. In our study, we correlated HER2 with Fak and further probed their roles in radioresistance in breast cancer for the several reasons. First, Fak expressed high in HER2 amplification breast cancer, and these two molecules are involved in ovarian cancer metastasis [27, 28]. Second, some studies show that HER2 amplification contributes to cell adhering ability and the anoikis inhibition of MCF-7 and MDA-MB-231 [29–31]. Fak is involved in these phenomena. Furthermore, accumulating evidence indicate that Fak promotes the EMT [32]. When HER2 is overexpressed, pFak increased significantly both in cell lines and tissue, although Fak remains stable. pFak decreased in the SK-BR-3 and ZR-7530 cell lines after HER2 silencing. Some studies suggest that HER2 might activate the EMT via TGF-B, and Fak is necessary in TGF-B induced EMT. Therefore, we conclude that HER2 might activate TGF-B, which would later lead to Fak activation. In animal experiments, we demonstrated that Smad2, p- Smad2, Smad3and pSmad3 expression increased in 231 PCDH HER2 mice compared with control mice. Smad4 is relatively stable. In these 4 cell lines, Smad2, pSmad2, Smad3 and Smad4 did not show obvious changes. However, pSmad3 increased significantly when HER2 was overexpressed. Similar variations were observed after HER2 silencing.

We used the Fak inhibitor PF-562271 [33, 34] and the solvent DMSO to treat MCF-7 HER2, HER2 231, and SK-BR-3 cells. The results showed that previously favorable clone survival with HER2 overexpression disappeared in breast cancer cells after Fak inhibition. A survival fraction of clones was recovered in MCF-7 HER2 and 231 HER2 cells after PF-562271 treatment. With respect to clone survival in SK-BR-3 cells after PF-562271 treatment, the effect was generally consistent with the effect in SK-BR-3 HER2i cells. The effect of PF-562271 indicates that Fak plays a critical role in HER2-overexpressing cells. This finding suggests that Fak inhibitors might serve as radiosensitizers for clinical applications. Although our study did not explore the radiosensitization effect of PF-562271 *in vivo*, small molecule Fak inhibitors have been applied in mouse models and have yielded good results in previous studies; these previous studies have shown that Fak inhibitors could suppress tumor growth, metastasis, and angiogenesis in mice [32, 35, 36]. PF-562271 (used in the current study) is an ATP-competitive inhibitor of Fak and has undergone a phase I clinical trial. The trial indicated that PF-562271 has good tolerance and safety and could stabilize disease progression. However, there have been no further phase II or III trials for PF-562271 because it exhibits nonlinear pharmacokinetic properties [33].

We demonstrated that HER2 could be a vital regulator in breast cancer radioresistance via Fak and phosphorylated Fak. HER2/Fak might be promising targets for breast cancer radiotherapy. More in depth and comprehensive studies might be needed to determine the correlation between Fak and HER2.

## MATERIALS AND METHODS

### Cell lines and cell culture

Human breast cancer cell lines MCF-7 (ATCC^®^ HTB-22™), ZR-75-30 (ATCC^®^ CRL-1504™), SK-BR-3 (ATCC^®^ HTB-30™), MDA-MB-231 (ATCC^®^ HTB-26™) and lentiviral packaging cell (293T cell) were purchased from American Type Culture Collection (Manassas, VA, USA). All cell lines were maintained in DMEM medium, supplemented with 10% fetal bovine serum, penicillin (100 units/mL), and streptomycin (100 μg/mL). All cell cultures were incubated at 37°C in a 5% CO_2_ atmosphere.

### Chemicals

PF-562271, a Fak inhibitor [34], was purchased from Selleck Chemicals (Houston, USA) and dissolved in DMSO. The final DMSO concentrations of the solution used throughout the study did not exceed 0.1%. Cells were grown to 70–80% confluence on plates and then were treated with different concentrations of PF-562271 for 24 h.

### Cloning of HER2 cDNA and transfection

RNA isolated from SK-BR-3 cells was used for reverse transcription with the PrimeScript™ 1st Strand cDNA Synthesis Kit (TaKaRa, Kusatsu, Japan) according to the protocol. The in-frame coding region of HER2 was PCR-amplified and inserted into the *NheI* and *NotI* sites of the pCDH-CMV-MCS-EF1-Puro vector. The forward primer was 5′-aattGCTAGCGCCACCatggagctggcggccttgt-3′; the reverse primer is 5′-ataagaatGCGGCCGCtcacactggcacgt ccagac-3′. The sequence of the HER2 insert was verified by DNA sequencing. A short hairpin RNA (shRNA) against HER2 was constructed to pLKO.1 puro plasmid according to the protocol available on the website (http://www.addgene.org/tools/protocols/plko/). The shRNA sequences targeting HER2 were referred to TRCN0000039878: forward: 5′-CCGGTGTCAGTATCCA GGCTTTGTACTCGAGTACAAAGCCTGGATACTGA CATTTTTG-3′; reverse: 5′-AATTCAAAAATGTCAGT ATCCAGGCTTTGTACTCGAGTACAAAGCCTGGATA CTGACA-3′. Restriction enzymes were purchased from New England Biolabs (Ipswitch, MA, USA), and T4 DNA ligase was from Promega (Madison, Wisconsin, USA). Primers were synthesized by Sangon Biotech (Shanghai, China). Lentiviruses expressing HER2 cDNA or HER2 shRNA and their corresponding empty vectors were produced by the transfection of plasmids into 293T cells and were used to infect target cells by using a method described previously. The cells were selected with puromycin (1.5 μg/mL) for 10–14 days.

### Real-time PCR

Total RNA was isolated from 2 × 10^6^ target cells using the Trizol reagent (Invitrogen, Carlsbad, CA). cDNAs generated by reverse transcription were used for real-time PCR with the ExScript RT-PCR kit (TaKaRa, Kusatsu, Japan). The qPCR primers for HER2 are as follows: 5′-CCCATATGTCTCCCGCCTTC-3′ (sense) and 5′-GGTTTTCCCGGACATGGTCT-3′ (antisense). The qPCR primers for GAPDH are as follows: 5′- ACCCAGAAGACTGTGGATGG-3′ (sense) and 5′-TCTAGACGGCAGGTCAGGTC-3′ (antisense). All amplifications and detections were carried out in the LightCycler 480 system (Roche, Basel, Switzerland) using the LightCycler 480 SYBR Green I Master (Roche, Basel, Switzerland). The statistical analyses were performed using the 2^−*#x0394;ΔCt^ relative quantification method.

### Cell proliferation assay

A total of 2.0 × 10^3^ cells per well were incubated in 96-well culture plates in 100 μl medium. The cells were incubated for 12 h to allow for attachment, after which a 0-time point measurement was determined. After culturing for 1, 2, 3, 4, and 5 days, the supernatant was removed, and cell growth was detected using the Cell Counting Kit-8(CCK-8) (Dojindo Laboratories, Kumamoto, Japan) according to the manufacturer's instructions. The absorbance at 450 nm was measured using a microplate reader. All proliferation assays were performed independently at least 3 times.

### Cell adhesion assay

ECM-coated plates were removed from the refrigerator and placed at room temperature under sterile conditions for 10 min. After conventional digestion and PBS washing, the cells were re-suspended in serum-free medium, and the cell concentration was adjusted to 1.0 ×10^5^−1.0 × 10^6^ cells/ml. A 150 μl aliquot of the cell suspension was added to the ECM-coated plates. The plates were incubated in an incubator for 30–90 minutes. The medium was carefully removed (without drying the wells), and the plates were gently washed with 250 μl of PBS 3–4 times. The PBS was aspirated from each well, and 200 μl of the cell staining solution was added for 10 min of staining at room temperature. The staining solution was then discarded, and the cells were gently washed 4–5 times with 500 μl of deionized water. The deionized water was then discarded, and the wells were dried at room temperature. A 200 μl aliquot of lysis buffer was added to each well, and the plates were incubated for 10 min on a slow shaker. A 150 μl aliquot of the lysate was transferred to 96-well plates, and the absorbance of the cell lysates at a wavelength of 560 nm was measured on a microplate reader.

### Anoikis assay

Cells (3.0 × 10^5^ −5.0 × 10^5^) were suspension-cultured in ultra-low-attachment six-well plates for 24–48 h. An equal (or smaller) number of cells were cultured in normal-attachment six-well plates for the same period. The cells were harvested at the designated time points, and the percentage of apoptotic cells was determined by Annexin V/PI double staining. The cells were digested and counted, followed by centrifugation at 1,800 rpm for 5 min. After two washes with pre-cold PBS, the cells were re-suspended with 1 × binding buffer. Then, 100 μl (1 × 10^5^ cells) aliquots of the cell suspension were transferred into 5-ml flow tubes, followed by the addition of 5 μl of FITC-labeled Annexin V and 5 μl of PI. The reaction was gently mixed and incubated in the dark at room temperature for 15 min. Then, 400 μl of 1× binding buffer was added to each tube. The flow cytometry assay was completed within 1 h.

### Irradiation and colony formation assay

Exponential growth phase cells were irradiated with X-rays (0, 2, 4, 6, and 8 Gy), using a linear accelerator (Varian Medical Systems, Palo Alto, CA) at 3 Gy/min and then digested for counting. The cell inoculum of the single cell suspension was determined for each sample according to the expected number of colony formation (30–100). The cells were seeded into 60-mm plastic petri dishes, and the dishes were incubated for 10 to 14 days to allow colonies to develop. The colonies were fixed with 4% paraformaldehyde and stained with 0.1% crystal violet (100% methanol solution) before being counted. The number of clones containing ≥ 50 cells was counted under a stereomicroscope. The plating efficiency (PE) was calculated as follows: PE = the number of colonies formed without irradiation/the number of cells inoculated × 100%. The survival fraction (SF) of cells was calculated at each irradiation dose as follows: SF = the number of colonies formed at a certain irradiation dose/(the number of cells inoculated × PE). All assays were performed independently at least 3 times.

### Western blotting

Cell lysates were prepared at 75% of confluence by using 500 μL of radio-immunoprecipitation assay buffer (25 mM Tris-HCl at pH 7.6, 150 mM NaCl, 1% Nonidet P-40, 1% sodium deoxycholate, and 0.1% sodium dodecyl sulfate) in 10-cm culture dishes with a 20-minute incubation on ice. The protein concentrations of the lysates were measured with a Bio-Rad protein assay kit (Hercules, CA). Immunoblot analyses were performed as previously described [37, 38]. β-Actin antibodies were obtained from Santa Cruz Biotechnology. HER2, Claudin-1, ZEB1, ZO-1, Fak, pFak, Smad, pSmad were purchased from Cell Signaling Technology. The secondary antibodies were the F(ab)_2_ fragment of donkey anti-mouse immunoglobulin (product NA931) or of donkey anti-rabbit immunoglobulin (product NA9340) linked to horseradish peroxidase from Amersham Biosciences (Little Chalfont, Buckinghamshire, UK). The immunoblot reagents were from an electrochemiluminescence kit (Amersham Biosciences, NJ, USA).

### *In vivo* research

The animal experiments were approved by the Experimental Animal Ethics Committee of Fudan University Shanghai medical college. Tumor inoculation assays in nude mice have been described previously [37–38]. Briefly, a total of 4 × 10^6^ −1 × 10^7^ 231 PCDH cells or 231 PCDH HER2 cells suspended in 100 μl of sterile phosphate-buffered saline (PBS) were injected subcutaneously into the right hind legs of 24 male BALB/c athymic mice. The tumor volumes were calculated with the following formula: tumor volume (mm3) = a × b^2^ × 0.50 (a represents the longest diameter of the tumor, b represents the shortest diameter of the tumor, and 0.50 is an empirical constant) [39]. When the tumors reached 0.5 cm^3^ in volume, the tumor areas in 12 mice (6 mice, 231 PCDH; 6 mice, 231 PCDH HER2) were irradiated with 6-MV X-rays (12 Gy, single fraction) and followed for approximately 4 weeks, and then the experiments were terminated. Xenograft tumors were flash frozen in liquid nitrogen and then stored at −80°C or proceeded to direct preparation of the samples. Frozen tissue (present in liquid nitrogen) was broken into several small pieces. The tissue was smashed into smaller pieces in dry ice, and then lysis buffer 2.5 μl/mg was added. The tissue was sonicated for 5 sec at a low setting on ice and then incubated on ice for 30 min. The samples were spun at 13,000 rpm for 15 min at 4°C.

### Statistical analysis

The statistical analysis was performed using SPSS Statistics 22.0 (IBM SPSS, Somers, NY, USA). The cell count, apoptosis rate, survival rate, survival fraction, and relative expression of associated genes and proteins were expressed as the mean ± standard deviation (SD)/standard error (SEM). Comparisons between multiple were conducted using a one-way analysis of variance. Pairwise comparisons were performed using the least significant difference *t*-test. The experimental data were analyzed and plotted using GraphPad Prism 6.0 (GraphPad Software Inc., San Diego, CA, USA). *P* value less than 0.05 was considered statistically significant.

## References

[1] Jia M, Zheng R, Zhang S, Zeng H, Zou X, Chen W (2015). Female breast cancer incidence and mortality in 2011, China. J Thorac Dis.

[2] Darby S, McGale P, Correa C, Taylor C, Arriagada R, Clarke M, Cutter D, Davies C, Ewertz M, Godwin J, Gray R, Pierce L, Whelan T (2011). Effect of radiotherapy after breast-conserving surgery on 10-year recurrence and 15-year breast cancer death: meta-analysis of individual patient data for 10,801 women in 17 randomised trials. Lancet.

[3] McGale P, Taylor C, Correa C, Cutter D, Duane F, Ewertz M, Gray R, Mannu G, Peto R, Whelan T, Wang Y, Wang Z, Darby S (2014). Effect of radiotherapy after mastectomy and axillary surgery on 10-year recurrence and 20-year breast cancer mortality: meta-analysis of individual patient data for 8135 women in 22 randomised trials. Lancet.

[4] Fisher B, Anderson S, Bryant J, Margolese RG, Deutsch M, Fisher ER, Jeong JH, Wolmark N (2002). Twenty-year follow-up of a randomized trial comparing total mastectomy, lumpectomy, and lumpectomy plus irradiation for the treatment of invasive breast cancer. N Engl J Med.

[5] Fisher B, Jeong JH, Anderson S, Bryant J, Fisher ER, Wolmark N (2002). Twenty-five-year follow-up of a randomized trial comparing radical mastectomy, total mastectomy, and total mastectomy followed by irradiation. N Engl J Med.

[6] Cheang MC, Chia SK, Voduc D, Gao D, Leung S, Snider J, Watson M, Davies S, Bernard PS, Parker JS, Perou CM, Ellis MJ, Nielsen TO (2009). Ki67 index, HER2 status, and prognosis of patients with luminal B breast cancer. J Natl Cancer Inst.

[7] Perou CM, Sorlie T, Eisen MB, van de Rijn M, Jeffrey SS, Rees CA, Pollack JR, Ross DT, Johnsen H, Akslen LA, Fluge O, Pergamenschikov A, Williams C (2000). Molecular portraits of human breast tumours. Nature.

[8] Voduc KD, Cheang MC, Tyldesley S, Gelmon K, Nielsen TO, Kennecke H (2010). Breast cancer subtypes and the risk of local and regional relapse. J Clin Oncol.

[9] Ribelles N, Perez-Villa L, Jerez JM, Pajares B, Vicioso L, Jimenez B, de Luque V, Franco L, Gallego E, Marquez A, Alvarez M, Sanchez-Munoz A, Perez-Rivas L (2013). Pattern of recurrence of early breast cancer is different according to intrinsic subtype and proliferation index. Breast Cancer Res.

[10] Park S, Koo JS, Kim MS, Park HS, Lee JS, Lee JS, Kim SI, Park BW (2012). Characteristics and outcomes according to molecular subtypes of breast cancer as classified by a panel of four biomarkers using immunohistochemistry. Breast.

[11] Sotiriou C, Neo SY, McShane LM, Korn EL, Long PM, Jazaeri A, Martiat P, Fox SB, Harris AL, Liu ET (2003). Breast cancer classification and prognosis based on gene expression profiles from a population-based study. Proc Natl Acad Sci U S A.

[12] Paik S, Kim C, Wolmark N (2008). HER2 status and benefit from adjuvant trastuzumab in breast cancer. N Engl J Med.

[13] Slamon D, Eiermann W, Robert N, Pienkowski T, Martin M, Press M, Mackey J, Glaspy J, Chan A, Pawlicki M, Pinter T, Valero V, Liu MC (2011). Adjuvant trastuzumab in HER2-positive breast cancer. N Engl J Med.

[14] Buchholz TA, Huang EH, Berry D, Pusztai L, Strom EA, McNeese MD, Perkins GH, Schechter NR, Kuerer HM, Buzdar AU, Valero V, Hunt KK, Hortobagyi GN (2004). Her2/neu-positive disease does not increase risk of locoregional recurrence for patients treated with neoadjuvant doxorubicin-based chemotherapy, mastectomy, and radiotherapy. Int J Radiat Oncol Biol Phys.

[15] Slack-Davis JK, Hershey ED, Theodorescu D, Frierson HF, Parsons JT (2009). Differential requirement for focal adhesion kinase signaling in cancer progression in the transgenic adenocarcinoma of mouse prostate model. Mol Cancer Ther.

[16] Wendt MK, Schiemann WP (2009). Therapeutic targeting of the focal adhesion complex prevents oncogenic TGF-beta signaling and metastasis. Breast Cancer Res.

[17] Lazaro G, Smith C, Goddard L, Jordan N, McClelland R, Barrett-Lee P, Nicholson RI, Hiscox S (2013). Targeting focal adhesion kinase in ER+/HER2+ breast cancer improves trastuzumab response. Endocr Relat Cancer.

[18] Lowery AJ, Kell MR, Glynn RW, Kerin MJ, Sweeney KJ (2012). Locoregional recurrence after breast cancer surgery: a systematic review by receptor phenotype. Breast Cancer Res Treat.

[19] Swain SM, Baselga J, Kim SB, Ro J, Semiglazov V, Campone M, Ciruelos E, Ferrero JM, Schneeweiss A, Heeson S, Clark E, Ross G, Benyunes MC (2015). Pertuzumab, trastuzumab, and docetaxel in HER2-positive metastatic breast cancer. N Engl J Med.

[20] Cao N, Li S, Wang Z, Ahmed KM, Degnan ME, Fan M, Dynlacht JR, Li JJ (2009). NF-kappaB-mediated HER2 overexpression in radiation-adaptive resistance. Radiat Res.

[21] Duru N, Fan M, Candas D, Menaa C, Liu HC, Nantajit D, Wen Y, Xiao K, Eldridge A, Chromy BA, Li S, Spitz DR, Lam KS (2012). HER2-associated radioresistance of breast cancer stem cells isolated from HER2-negative breast cancer cells. Clin Cancer Res.

[22] Al-Hajj M, Wicha MS, Benito-Hernandez A, Morrison SJ, Clarke MF (2003). Prospective identification of tumorigenic breast cancer cells. Proc Natl Acad Sci U S A.

[23] Giordano A, Gao H, Anfossi S, Cohen E, Mego M, Lee BN, Tin S, De Laurentiis M, Parker CA, Alvarez RH, Valero V, Ueno NT, De Placido S (2012). Epithelial-mesenchymal transition and stem cell markers in patients with HER2-positive metastatic breast cancer. Mol Cancer Ther.

[24] Arias-Romero LE, Villamar-Cruz O, Huang M, Hoeflich KP, Chernoff J (2013). Pak1 kinase links ErbB2 to beta-catenin in transformation of breast epithelial cells. Cancer Res.

[25] Schade B, Lesurf R, Sanguin-Gendreau V, Bui T, Deblois G, O'Toole SA, Millar EK, Zardawi SJ, Lopez-Knowles E, Sutherland RL, Giguere V, Kahn M, Hallett M (2013). beta-Catenin signaling is a critical event in ErbB2-mediated mammary tumor progression. Cancer Res.

[26] Oliveras-Ferraros C, Corominas-Faja B, Cufi S, Vazquez-Martin A, Martin-Castillo B, Iglesias JM, Lopez-Bonet E, Martin AG, Menendez JA (2012). Epithelial-to-mesenchymal transition (EMT) confers primary resistance to trastuzumab (Herceptin). Cell Cycle.

[27] Villa-Moruzzi E (2013). PTPN12 controls PTEN and the AKT signalling to FAK and HER2 in migrating ovarian cancer cells. Mol Cell Biochem.

[28] Aponte M, Jiang W, Lakkis M, Li MJ, Edwards D, Albitar L, Vitonis A, Mok SC, Cramer DW, Ye B (2008). Activation of platelet-activating factor receptor and pleiotropic effects on tyrosine phospho-EGFR/Src/FAK/paxillin in ovarian cancer. Cancer Res.

[29] Cordes N, Meineke V (2003). Cell adhesion-mediated radioresistance (CAM-RR). Extracellular matrix-dependent improvement of cell survival in human tumor and normal cells *in vitro*. Strahlenther Onkol.

[30] Paoli P, Giannoni E, Chiarugi P (2013). Anoikis molecular pathways and its role in cancer progression. Biochim Biophys Acta.

[31] Demers MJ, Thibodeau S, Noel D, Fujita N, Tsuruo T, Gauthier R, Arguin M, Vachon PH (2009). Intestinal epithelial cancer cell anoikis resistance: EGFR-mediated sustained activation of Src overrides Fak-dependent signaling to MEK/Erk and/or PI3-K/Akt-1. J Cell Biochem.

[32] Wilson C, Nicholes K, Bustos D, Lin E, Song Q, Stephan JP, Kirkpatrick DS, Settleman J (2014). Overcoming EMT-associated resistance to anti-cancer drugs via Src/FAK pathway inhibition. Oncotarget.

[33] Infante JR, Camidge DR, Mileshkin LR, Chen EX, Hicks RJ, Rischin D, Fingert H, Pierce KJ, Xu H, Roberts WG, Shreeve SM, Burris HA, Siu LL (2012). Safety, pharmacokinetic, and pharmacodynamic phase I dose-escalation trial of PF-00562271, an inhibitor of focal adhesion kinase, in advanced solid tumors. J Clin Oncol.

[34] Stokes JB, Adair SJ, Slack-Davis JK, Walters DM, Tilghman RW, Hershey ED, Lowrey B, Thomas KS, Bouton AH, Hwang RF, Stelow EB, Parsons JT, Bauer TW (2011). Inhibition of focal adhesion kinase by PF-562,271 inhibits the growth and metastasis of pancreatic cancer concomitant with altering the tumor microenvironment. Mol Cancer Ther.

[35] Taliaferro-Smith L, Oberlick E, Liu T, McGlothen T, Alcaide T, Tobin R, Donnelly S, Commander R, Kline E, Nagaraju GP, Havel L, Marcus A, Nahta R (2015). FAK activation is required for IGF1R-mediated regulation of EMT, migration, and invasion in mesenchymal triple negative breast cancer cells. Oncotarget.

[36] Hao H, Naomoto Y, Bao X, Watanabe N, Sakurama K, Noma K, Motoki T, Tomono Y, Fukazawa T, Shirakawa Y, Yamatsuji T, Matsuoka J, Wang ZG (2009). Focal adhesion kinase as potential target for cancer therapy [Review]. Oncol Rep.

[37] Hou J, Wang Z, Xu H, Yang L, Yu X, Yang Z, Deng Y, Meng J, Feng Y, Guo X, Yang G (2015). Stanniocalicin 2 suppresses breast cancer cell migration and invasion via the PKC/claudin-1-mediated signaling. PLoS One.

[38] Yang G, Thompson JA, Fang B, Liu J (2003). Silencing of H-ras gene expression by retrovirus-mediated siRNA decreases transformation efficiency and tumorgrowth in a model of human ovarian cancer. Oncogene.

[39] Marushige Y, Raju NR, Marushige K, Koestner A (1987). Modulation of growth and of morphological characteristics in glioma cells by nerve growth factor and glia maturation factor. Cancer Res.

